# Comparison between clinical grading and navigation data of knee laxity in ACL-deficient knees

**DOI:** 10.1186/1758-2555-2-27

**Published:** 2010-11-08

**Authors:** Yuji Yamamoto, Yasuyuki Ishibashi, Eiichi Tsuda, Harehiko Tsukada, Shugo Maeda, Satoshi Toh

**Affiliations:** 1 Department of Orthopaedic Surgery, Hirosaki University Graduate School of Medicine, Hirosaki, Aomori, Japan

## Abstract

**Background:**

The latest version of the navigation system for anterior cruciate ligament (ACL) reconstruction has the supplementary ability to assess knee stability before and after ACL reconstruction. In this study, we compared navigation data between clinical grades in ACL-deficient knees and also analyzed correlation between clinical grading and navigation data.

**Methods:**

150 ACL deficient knees that received primary ACL reconstruction using an image-free navigation system were included. For clinical evaluation, the Lachman, anterior drawer, and pivot shift tests were performed under general anesthesia and were graded by an examiner. For the assessment of knee stability using the navigation system, manual tests were performed again before ACL reconstruction. Navigation data were recorded as anteroposterior (AP) displacement of the tibia for the Lachman and anterior drawer tests, and both AP displacement and tibial rotation for the pivot shift test.

**Results:**

Navigation data of each clinical grade were as follows; Lachman test grade 1+: 10.0 mm, grade 2+: 13.2 ± 3.1 mm, grade 3+: 14.5 ± 3.3 mm, anterior drawer test grade 1+: 6.8 ± 1.4 mm, grade 2+: 7.4 ± 1.8 mm, grade 3+: 9.1 ± 2.3 mm, pivot shift test grade 1+: 3.9 ± 1.8 mm/21.5° ± 7.8°, grade 2+: 4.8 ± 2.1 mm/21.8° ± 7.1°, and grade 3+: 6.0 ± 3.2 mm/21.1° ± 7.1°. There were positive correlations between clinical grading and AP displacement in the Lachman, and anterior drawer tests. Although positive correlations between clinical grading and AP displacement in pivot shift test were found, there were no correlations between clinical grading and tibial rotation in pivot shift test.

**Conclusions:**

In response to AP force, the navigation system can provide the surgeon with correct objective data for knee laxity in ACL deficient knees. During the pivot shift test, physicians may grade according to the displacement of the tibia, rather than rotation.

## Background

The Lachman, anterior drawer, and pivot shift tests are clinical examinations commonly used to diagnose anterior cruciate ligament (ACL) injury or deficiency and also evaluate ACL-reconstructed knees at follow-up. Both the Lachman and anterior drawer tests evaluate the tibial translation in response to an anterior load applied to the tibia, and especially the Lachman test can measure quantitatively using an arthrometer such as a KT-1000 that determines the magnitude of movement in mm [1]. However, measurement in anterior tibial translation only may not be adequate to assess ACL-reconstructed knees because the presence of a positive pivot shift test was more predictive of later knee osteoarthritis, failure to return to previous sports level, and patient-reported poor outcome in function and symptom after ACL reconstruction [2-4].

The pivot shift test is conducted by simultaneously applying valgus and axial tibial torques to the knee at near extension and evaluating subluxation of the tibia while gradually flexing the knee [5,6]. With an ACL deficient knee, the lateral tibial plateau subluxes anteriorly when the knee is extended with the tibia in internal rotation but then suddenly reduces as the knee is flexed. This reduction of the lateral tibial plateau characterizes a positive pivot shift test and has been described as a sudden decrease in anterior tibial translation and internal tibial rotation during knee flexion [7-9]. Therefore, the multiple degrees of freedom knee kinematics may be monitored to understand pivot shift phenomenon. However, the pivot shift test is clinically graded based on the physician's subjective feeling of the movement of the tibia and also may be difficult to be quantitatively evaluated by conventional instruments.

Recently, a computer-assisted navigation system has been introduced in ACL reconstruction to improve accuracy of bone tunnel placement [10-12]. In addition to assisting the surgeon to decide the proper tunnel position during surgery, the latest version of the navigation system has the supplementary ability to assess knee kinematics before and after ACL reconstruction during surgery [13-17]. Therefore, the navigation system could be one of the tools to quantitatively evaluate knee kinematics or laxity in ACL deficient knees. In this study, ACL deficient knees were graded by manual test of knee laxity under general anesthesia and the knee kinematics were also evaluated using the navigation system before ACL reconstruction. The purpose of this study was to compare navigation data between clinical grades of laxity in ACL-deficient knees and also analyze correlation between clinical grading and navigation data.

## Methods

### Subjects

From January 2006 to January 2009, 150 ACL deficient knees that received navigated primary ACL reconstruction with either hamstring tendon or bone-patellar tendon-bone autograft in our hospital were included in this study. There were 57 men and 92 women and patient ages ranged from 12 to 59 years (average age: 22.8 years). Only one patient had bilateral ACL injuries. Patients with other concomitant ligament injury such as PCL injury on physical examination or MRI were excluded. The study design was approved by the ethics committee in our institution, and all patients provided informed consent to participate.

### Clinical grading

For clinical evaluation of knee laxity in ACL deficient knees, manual tests, including the Lachman test, the anterior drawer test and the pivot shift test, were performed by a single orthopaedic surgeon (E. T.) under general anesthesia before ACL reconstruction. The result of each manual test was graded by the examiner using International Knee Document Committee criteria as grade 0, 1+, 2+, or 3+.

### Navigation process and data collection

The OrthoPilot navigation system (ACL version 2.0; B/Braun AESCULAP, Tuttlingen, Germany) was used in this study. This image-free, wireless system does not require preoperative computed tomography or intraoperative fluoroscopy. This version can provide the surgeon with knee kinematics, such as anterior-posterior (AP) displacement and internal-external rotation of the tibia as well as the intra-operative information, such as intra-articular position of tibial and femoral tunnels. The accuracy of this system is extremely precise and the cameras can track the position of the instruments to within < 1 mm and < 1° [11].

For the navigation process, the femoral and tibial transmitters were firmly secured to the femur or tibia by the fixation instruments with two K-wires each. Both anatomical landmarks and knee kinematics were registered. Anatomical landmarks consisted of the tibial tuberosity, the anterior edge of the tibia, and the medial and lateral point of the tibia plateau. The knee kinematics between 0° - 90° of knee flexion were registered. All navigation processes (registrations of anatomical landmarks and knee kinematics) and the following evaluation of the knee laxity using the navigation system were performed by a single surgeon (Y. I.).

Before ACL reconstruction, knee laxity tests were performed under the navigation system. Manual maximum AP forces were applied to the tibia in neutral rotation, and AP displacement of the tibia was measured at 30° and 90° of knee flexion by the Lachman test and the anterior drawer test, respectively. The pivot shift test was performed by applying valgus and internal torque to the knee. Maximum anterior displacement of the tibia and internal rotation angles from the initial external rotation position were measured at the knee flexion angle that the examiner felt the tibia was most displaced on the femur. The knee was then flexed further, and maximum anterior displacement and internal rotation of the tibia were similarly measured after reduction of the tibia occurred. Navigation data were recorded as AP displacement of the tibia for the Lachman test or anterior drawer test, and both AP displacement and tibial rotation for the pivot shift test.

### Statistical analysis

Multiple comparison procedure was performed for comparison of navigation data between clinical grades (SPSS 16.0; SPSS Science Inc, Chicago, IL). Spearman's rank correlation was used to detect the correlation in navigation data of different clinical grades. P values of < 0.05 were considered statistically significant.

## Results

### Clinical grading

None of the ACL deficient knees were graded as 0 in any of the manual tests under general anesthesia. The results of the Lachman test were grade 1+ in 1 patient, grade 2+ in 39 patients, and grade 3+ in 110 patients. The results of the anterior drawer test were grade 1+ in 10 patients, grade 2+ in 83 patients, and grade 3+ in 57 patients. The results of the pivot shift test were grade 1+ in 12 patients, grade 2+ in 87 patients, grade 3+ in 51 patients.

### Navigation data

In the Lachman and anterior drawer tests, navigation data showed that mean AP displacement was 14.2 ± 3.3 mm and 8.0 ± 2.1 mm at 30 and 90 degrees of flexion, respectively. In the pivot shift test, anterior displacement and internal rotation of the tibia before reduction were measured at an average of 19.1° ± 4.7° of knee flexion. Maximum anterior displacement and rotation of the tibia before reduction were 5.1 ± 2.6 mm/21.5° ± 7.1°. After reduction of the tibia during the pivot shift test, AP displacement and rotation of the tibia were measured at an average of 42.9° ± 5.2° of knee flexion. AP displacement of the tibia and internal rotation of the tibia were 3.3 ± 1.7 mm/26.0° ± 6.2°.

Navigation data of each clinical grade were as follows; the Lachman test grade 1+: 10.0 mm, grade 2+: 13.2 ± 3.1 mm, grade 3+: 14.5 ± 3.3 mm, the anterior drawer test grade 1+: 6.8 ± 1.4 mm, grade 2+: 7.4 ± 1.8 mm, grade 3+: 9.1 ± 2.3 mm (Table 1). In the higher clinical grades, the mean displacement measured using navigation increased significantly. In the pivot shift test, navigation data (displacement/rotation of the tibia) before reduction were grade 1+: 3.9 ± 1.8 mm/21.5° ± 7.8°, grade 2+: ± 2.1 mm/21.8° ± 7.1°, grade 3+: 6.0 ± 3.2 mm/21.1° ± 7.1° (Table 1). After reduction of the tibia, navigation data were grade 1+: 3.4 ± 1.6 mm/25.6° ± 7.2°, grade 2+: 3.1 ± 1.5 mm/26.3° ± 5.7°, grade 3+: 3.6 ± 1.9 mm/25.5° ± 6.8°. Significant differences between clinical grades were only found in displacement before reduction. Mean differences in displacement of the tibia before and after reduction were grade 1+: 0.7 ± 2.4 mm, grade 2+: 1.7 ± 2.2 mm, and grade 3+: 2.4 ± 2.8 mm, and there were no significant differences between them.

**Table 1:** Navigation data of each clinical grade

	Clinical grade		
	
	Grade 1+	Grade 2+	Grade 3+
**Lachman test**			
Displacement (mm)	10.0	13.2 ± 3.1^c^	14.5 ± 3.3^b^
**Anterior drawer test**			
Displacement (mm)	6.8 ± 1.4	7.4 ± 1.8^c^	9.1 ± 2.3^a, b^
**Pivot shift test**			
Before reduction			
Displacement (mm)	3.9 ± 1.8^c^	4.8 ± 2.1^c^	6.0 ± 3.2^a, b^
Rotation (deg)	21.5 ± 7.8	21.8 ± 7.1	21.1 ± 7.1
After reduction			
Displacement (mm)	3.4 ± 1.6	3.1 ± 1.5	3.6 ± 1.9
Rotation (deg)	25.6 ± 7.2	26.3 ± 5.7	25.5 ± 6.8

Data expressed as mean ± SD.

^a ^P < .05 compared to grade 1+.

^b ^P < .05 compared to grade 2+.

^c ^P < .05 compared to grade 3+.

### Correlation between clinical grading and navigation data

**Figure 1:**
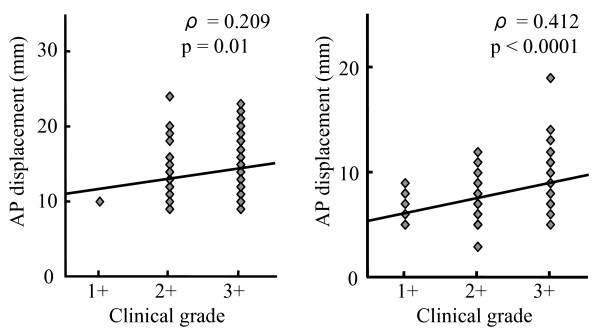
**Correlation between clinical grading and navigation data in the Lachman test (A) and the anterior drawer test (B)**.

**Figure 2:**
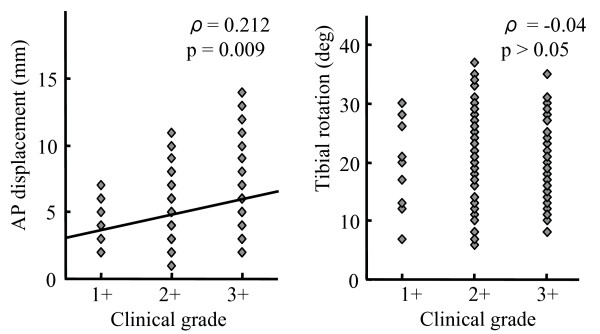
**Correlation between clinical grading and navigation data in the pivot shift test: (A) AP displacement, (B) Tibial rotation**.

Correlation analysis showed there were positive correlations between clinical grading and AP displacement in the Lachman (ρ = 0.209, p = 0.01), and anterior drawer tests (ρ = 0.412, p < 0.0001) (Figure 1). Although positive correlations between clinical grading and AP displacement before reduction in the pivot shift test were found (ρ = 0.212, p = 0.009), there were no correlations between clinical grading and tibial rotation before reduction in pivot shift test (ρ = -0.04, p = 0.620) (Figure 2). In terms of differences in displacement of the tibia before and after reduction, there was no correlation with clinical grading (ρ = 0.143, p = 0.080). Also, there were no correlations between clinical grading and difference in tibial rotation before and after reduction in pivot shift test (ρ = -0.032, p = 0.701).

## Discussion

In this study, knee laxity measured using a navigation system was compared between clinical grades in ACL-deficient knees, and correlations between clinical grade and navigation data were also analyzed. Essentially, the navigation system for ACL reconstruction has been a tool for increasing the precision of surgical procedure, especially bone tunnel placement. Recently, there were several publications which reported knee kinematics measured using the navigation system in ACL-deficient or ACL-reconstructed knees during surgery [13-20]. The navigation system we used in this study can evaluate accurately the AP displacement and the internal-external rotation of the tibia with respect to the femur at a selected angle of knee flexion in response to externally applied load.

In response to AP load such as the Lachman test and the anterior drawer test, navigation indicated that mean AP displacement was 14.2 ± 3.3 mm and 8.0 ± 2.1 mm at 30 and 90 degrees of flexion, respectively, in this study. Daniel et al. reported that 89 ACL injured knees had a mean anterior displacement of 13.0 mm at 30 degrees of flexion when using KT-2000 with 89 newtons [1]. Song et al. measured AP laxity with manual maximal force in 41 ACL injured knees using the same navigation system as we used in this study [19]. They reported that mean anterior displacements were 14.7 ± 3.5 and 8.5 ± 4.0 mm at 30 and 90 degrees of flexion, respectively. Therefore, results in this study were consistent with previous published works.

With regard to the Lachman test and the anterior drawer test, in the higher clinical grades, the mean displacement of the tibia measured using navigation increased significantly. Furthermore, there were positive correlations between clinical grading and AP displacement of the tibia during both tests. Therefore, the navigation system could provide surgeons with correct objective data for knee laxity in response to AP force.

The knee kinematics during the pivot shift test are difficult to quantify in the clinical setting. In biomechanical cadaveric studies, so-called simulated pivot shift tests were performed using robotic testing systems to measure multiple degrees of freedom knee kinematics in response to controlled combined rotatory loads [21-24].

Some in vivo studies tried to elucidate knee kinematics of ACL-deficient knees during pivot shift test and also to analyze correlation with clinical grading. Okazaki et al. quantified the anterolateral rotarory instability of ACL-deficient knees using an open MRI [25]. With the same measurement using open MRI, Tashiro et al. demonstrated that side-to-side difference of anterior displacement at the lateral compartment correlated with clinical grade of the pivot shift test [26]. Bull et al. demonstrated the knee kinematics during pivot shift test intraoperatively using an electromagnetic device [27]. They concluded that the pivot shift was most consistently described as a translation of the tibial plateau, rather than a rotation. Meanwhile, Hoshino et al. also evaluated pivot shift test using an electromagnetic measurement system [28]. Because the coupled tibial anterior translation and acceleration of posterior translation in the ACL-deficient knee were larger in correlation with clinical grading, they suggested that not only 3-dimensional position displacement but also 3-dimensional acceleration should be measured for quantitative evaluation of the pivot shift test. Lately, navigation systems have been used to quantify in vivo pivot shift phenomenon [13,29,30]. Lane et al. determined that tibial rotation, anterior tibial translation, acceleration of posterior translation, and the angle of P were distinct components of the pivot shift that predict clinical grade [29]. Lopomo et al. evaluated the area included by the curves describing AP translation during pivot shift test as an index of dynamic joint instability, and found that it was correlated with preoperative pivot shift grade [30].

In this study, during pivot shift testing, significant differences between clinical grades were only found in AP displacement before reduction. Although AP displacement before reduction positively correlated with clinical grading in the pivot shift test, there were no correlations between clinical grading and tibial rotation. Therefore, AP displacement of the tibia is responsible for the different clinical grades of pivot shift test. The navigation system we used could not evaluate dynamic or sudden movement of the knee such as pivot shift phenomenon, even though displacement and rotation of the tibia were measured in a static condition before or after reduction of the tibia during the pivot shift test. There were other limitations in this study. First, applied forces were not constant in both manual test and measurement using the navigation system. Second, correlation analyses provided positive statistical values, but they showed poor correlations. This was probably due to the use of a discrete grade for clinical grading of knee laxity. Finally, there was no data of intact knees because navigation has the disadvantage of requiring invasive rigid fixation of transmitters to the bone.

Although this study had theses limitations, results clearly showed that navigation data correlated with clinical grading of the Lachman and the anterior drawer tests, and tibial rotation did not correlate with clinical grading of the pivot shift but displacement of the tibia did. Therefore, physicians may grade according to the displacement of the tibia, rather than rotation during the pivot shift test. A newer version of the navigation system will be needed to understand in detail dynamic movement of the knee during pivot shift test.

## Conclusions

In the Lachman and anterior drawer test in ACL deficient knees, positive correlations between clinical grading and navigation data were confirmed. Therefore, the navigation system could provide surgeons with correct objective data for knee laxity in response to AP force. In the pivot shift test, clinical evaluations were not correlated with tibial rotation but displacement of the tibia. During pivot shift testing, physicians may grade according to the displacement of the tibia, rather than rotation.

## Competing interests

The authors declare that they have no competing interests.

## Authors' contributions

YY participated in data collection, conducted statistical analyses, and drafted the manuscript. YI conceived the main idea, participated in the design of the study, and its revision and coordination. ET participated in the development of the study question and in data collection. HT and SM participated in data collection, and in the analysis and the interpretation of data. ST participated in the revision of the manuscript. All authors read and approved the final manuscript.
